# Editorial: New Advances in Cardiorenal Syndrome

**DOI:** 10.3389/fcvm.2022.976846

**Published:** 2022-07-14

**Authors:** Laurent Calvier, Gábor Kökény, Ernesto Martinez-Martinez

**Affiliations:** ^1^Department of Molecular Genetics, University of Texas Southwestern Medical Center, Dallas, TX, United States; ^2^Center for Translational Neurodegeneration Research, University of Texas Southwestern Medical Center, Dallas, TX, United States; ^3^Institute of Translational Medicine, Semmelweis University, Budapest, Hungary; ^4^International Nephrology Research and Training Center, Semmelweis University, Budapest, Hungary; ^5^Departamento de Fisiología, Facultad de Medicina, Instituto de Investigación Sanitaria Gregorio Marañón, Universidad Complutense de Madrid, Madrid, Spain; ^6^Ciber de Enfermedades Cardiovasculares, Instituto de Salud Carlos III, Madrid, Spain

**Keywords:** cardiorenal syndrome, cardiovascular system, heart failure, kidney failure, inflammation, fibrosis, mitochondria, biomarker

Crosstalk between cardiovascular system and kidney becomes apparent when failure in one system adversely impacts the other in a negative loop. The term cardiorenal syndrome became more popular in the past 10 years and commonly refers to the collective dysfunction of heart and kidney resulting in a cascade of feedback mechanism causing damage to both organs and is associated with adverse clinical outcomes. Indeed, a large number of patients with acute decompensated heart failure present with various degrees of heart and kidney dysfunction.

Recently, cardiorenal syndrome has been categorized into 5 clinical subtypes that reflect the pathophysiology, the time frame, and the nature of concomitant cardiac and renal dysfunction (1). This clinical classification has raised interest on this condition, but its pathophysiology remains unclear and involves complex, multifactorial, and dynamic mechanisms. Some features are common in both heart failure and chronic kidney diseases, such as oxidative stress, inflammation and fibrosis leading to organ remodeling and dysfunction (2).

To study this syndrome, some mouse and rat models are available in which both cardiac and renal dysfunction are induced (3), but the translation of the results from animal studies to clinic is limited, highlighting the need for better models. In addition to rodent models and to circumvent interspecies limitations, Gabbin et al. reviewed *in vitro* options and advances mimicking the dynamic organ-organ crosstalk. For example, human induced pluripotent stem cells in combination with microfluidic chips represents a powerful tool with potential to recapitulate the characteristics of cardiorenal syndrome *in vitro*, thus offering a platform for therapy development. Human cohorts, animal models and human cell cultures together offer complementary approaches to study how complex hemodynamic, biochemical and hormonal factors contribute to the development of the cardiorenal syndrome. Often neglected in this heart-kidney crosstalk, mitochondria rapidly sense and respond to a wide range of stress stimuli. Shi et al. reviewed *in vitro* and animal studies that have identified an important role of mitochondrial dysfunction in heart failure and chronic kidney disease. They also discuss the current research evidence supporting that mitochondrial dysfunction is involved in various types of cardiorenal syndrome.

The communication between the heart and kidneys mainly relies on hormones, cytokines and other biomarkers circulating in the blood. Consequently, efforts have been made to identify those markers and use them for diagnostic and treatment (1). Following this idea, Caravaca Perez et al. have associated Galectin-3, a well-known inflammatory marker (4–10), with high mortality in patients with acute HF and renal dysfunction. They also demonstrated that renal function influences the prognostic value of Galectin-3 levels, which should be adjusted by eGFR (estimated glomerular filtration rate) for a correct interpretation. Besides plasma or serum, urines biomarkers can be also measured as demonstrated by Diaz-Riera et al., using the urinary vitamin D binding protein (uVDBP), a multifunctional protein with major functions as binding/transport for all vitamin D metabolites but also fatty acid transport, and immune system (11). This protein is increased in patients with acute decompensated heart failure at hospital admission and presents a differential evolution pattern at early stage of renal dysfunction, before pathological worsening of glomerular filtration rate is evidenced.

In addition to biomarkers, clinical parameters are followed and used to evaluate the course of the disease. For example, Lai et al. suggested that chronic kidney disease is associated with an increased risk of reduced ejection fraction (HFrEF), which was related to higher all-cause mortality in patients with coronary artery disease undergoing percutaneous coronary intervention. In another study, Palazzuoli et al. as for them proposed that chronic kidney disease severity is related to adverse event occurrence, however, its impact may differ among different renal function trajectories and subtypes during hospitalization. Consequently, persistent deterioration and transient improvement of renal function appear to be the two patterns associated with increased risk.

Because it is a complex multifactorial disease, our knowledge on the cardiorenal syndrome is constantly improved, and so are our therapeutic strategies. Thereby, Minh et al. point that despite their widespread use in the congestive heart failure, the evidence for intravenous loop diuretic administration is not as robust as many other treatments for heart failure and its advantages may be outweighed by the substantial risk of electrolyte disturbances and worsening renal function. Further, there is no consensus on the time point for early starting of add-on therapy and for the preferred diuretic combination. In their design and rationale paper, the authors lay out a new clinical study, the diuretic resistance acute heart failure (DR-AHF) trial, to demonstrate the efficacy of the timely intense loop diuretic monotherapy and tolvaptan combination treatment in the early stage of congestive heart failure with renal dysfunction and provide the clinical evidence of diuretic resistance.

This collection of publications offers a sample of advances in the field of cardiorenal syndrome, from bench side to bed side. It covers cell culture models, cellular response to stress, biomarkers, clinical parameters and improvement of clinical practice. Further collections or review are still needed to improve our comprehension of this syndrome. For example, to cover the discovery and the biology of biomarkers emerging in related fields (4, 6, 12–17), which would help to better stratify the patients and propose tailored therapies; or to explore repositioning of known treatments in related cardio/vascular/kidney diseases (18–22), which would allow fast deployment and increase therapeutic options for patients.

## Author contributions

LC wrote the editorial. GK and EM-M have revised it for interpretation and content. All authors contributed to the article and approved the submitted version.

## Conflict of interest

LC is a co-shareholder of Reelin Therapeutics Inc. and co-inventor of a patent related to anti-Reelin strategies (Application Number: 15/763,047 and Publication Number: 20180273637). The remaining authors declare that the research was conducted in the absence of any commercial or financial relationships that could be construed as a potential conflict of interest.

## Publisher's note

All claims expressed in this article are solely those of the authors and do not necessarily represent those of their affiliated organizations, or those of the publisher, the editors and the reviewers. Any product that may be evaluated in this article, or claim that may be made by its manufacturer, is not guaranteed or endorsed by the publisher.
